# Parental feeding practices data in healthy children and children with gastrointestinal diseases

**DOI:** 10.1016/j.dib.2020.106036

**Published:** 2020-07-17

**Authors:** Katerina Sdravou, Athanasia Printza, Elias Andreoulakis, Fotini Sotiriadou, Athanasios Evangeliou, Maria Fotoulaki

**Affiliations:** a 4th Department of Pediatrics, School of Medicine, Aristotle University of Thessaloniki, Ring Road, N. Eukarpia, Thessaloniki, Greece; b 1st Otolaryngology Department, School of Medicine, Aristotle University of Thessaloniki, Greece; c Department of Thessaloniki, Adult Psychiatric Unit, Hellenic Centre for Mental Health and Research, Greece

**Keywords:** Feeding problems, Dysphagia, Parental feeding practices, Gastrointestinal disease, Healthy children

## Abstract

Parental feeding practices significantly influence child eating behavior. The data for this article was from a cross-sectional case control larger study that aimed to record parental practices to manage feeding problems in children with typical development and children with gastrointestinal diseases. A set of 23 Likert-type questions was used to investigate parental practices. Demographic and anthropometric data were obtained via a structured set of questions. In total 765 parents of healthy children and 136 parents of children with gastrointestinal diseases aged one to seven years participated in the study. Healthy controls were recruited from kindergartens located in various geographical areas in Greece. Children with gastrointestinal diseases were recruited from a Pediatric Gastroenterology Outpatient Clinic. Descriptive measures (i.e. frequencies, percentages, means and standard deviations) alongside with statistical analysis measures are presented in this article. Chi-square tests and U-tests were performed for the purpose of the comparison between the two groups. Spearman's rho correlation coefficient was also calculated for inter-item correlations among the 23-items of the questionnaire.

**Specifications Table**

**Table:** 

**Subject**	Perinatology, Pediatrics and Child Health
**Specific subject area**	Parental practices to manage feeding problems
**Type of data**	Tables
**How data were acquired**	Parental feeding practices, parent and child demographics and child anthropometric data were obtained via structured set of questions.
**Data format**	Raw, analyzed.
**Parameters for data collection**	A set of 23 Likert-type questions was used to investigate parental feeding practices. Parent demographic data (sex, age, educational level and employment status), child demographic and anthropometric data (sex, age, presence of siblings, birth order, birth weight, current height and weight) were recorded. The current Body Mass Index (BMI) was calculated using WHO anthro and anthro-plus software.
**Description of data collection**	Children with gastrointestinal diseases aged between 1 and 7 years old were considered as potentially eligible for the study, as long as a gastrointestinal disease could be officially established. Children aged between 1 and 7 from various kindergartens and schools were approached and those that suffered from no chronic disease (i.e. were of typical development) were considered eligible.
**Data source location**	Greece 4th Department of Pediatrics, School of Medicine, Aristotle University of Thessaloniki, General Hospital “Papageorgiou”, Thessaloniki, Greece
**Data accessibility**	Mendeley depository Data identification number: DOI: 10.17632/c25bx2vpnw.1 Direct URL: 10.17632/c25bx2vpnw.1
**Related research article**	K. Sdravou, E. Andreoulakis, A. Printza, F. Sotiriadou, A. Evangeliou, M. Fotoulaki, Parental management of feeding problems in young children- a population-based study, Int. J. Pediatr. Otorhinolaryngol. 136 (2020) 110,162. 10.1016/j.ijporl.2020.110162

**Value of the Data**

• These data are useful because they describe parental practices when managing feeding problems and explore factors that may be associated with these practises.
• All researchers involved in child feeding can benefit from these data because they provide a thorough understanding of parental behavior during child feeding.
• Data can be used for further research on the management of feeding problems in children.
• Parental feeding practises data for this sample can be compared with that for other sample for further insight.
• Our data can be used to develop intervention programs aiming to address feeding problems in children.
• Further research could focus on the evaluation of the properties (reliability, validity, factorial structure to name the most fundamental) of this 23-item questionnaire (or a shorter version of it). The correlation matrix is provided for this purpose as an initial investigation step

## 1 Data description

Table 1 presents means and standard deviations of the raw score (ranged from 1–5) on each of the 23 items are provided alongside U test (and p) values to quantify between groups comparison. Table 2 shows frequencies of each level of the 5-likert scale answers (ranging from “never” to “always”) for all 23 items of the questionnaire alongside with chi-square test (and p) values to quantify between groups comparison. Table 3 presents a correlation matrix (Spearman's rho coefficient) of the 23-item questionnaire to depict inter-item relations (control group). Table 4 shows a correlation matrix (Spearman's rho coefficient) of the 23-item questionnaire to depict inter-item relations (clinical group). Appendix A depicts the 23-item questionnaire (supplementary material). Research data for this article can be found at 10.17632/c25bx2vpnw.1 [1].

**Table 1:** Between groups comparison concerning the mean score in each of the 23 items of the questionnaire.

	Healthy(Control) Group	Gastrointestinal disease(Clinical) Group	Mann-Whitney U test	p
	Mean ± SD	Mean ± SD		
1. I accept that he/she may not be hungry, and I take the food away.	3.43±1.00	3.60±1.04	47,796.5	0.112
2. I let the child take a break and try to feed him/her a little later.	2.64±1.09	2.90±1.21	46,202.0	**0.031**
3. I urge the child to eat with prompts such as: "eat at least a little", "please try to eat", "Do you want to try them? I prepared what you like” etc.	2.82±1.14	3.10±1.19	45,351.5	**0.014**
4. I urge the child to eat by saying for example: "the food will get cold" or "eat your beans fast" or "you can eat it" etc.	2.51±1.07	2.51±1.08	51,960.5	0.982
5. I ask my family or other people to encourage the child to eat.	1.75±0.95	2.17±1.26	42,991.0	**<0.001**
6. I say to my child that I or someone else in the family is eating (e.g., "I am eating," or "your brother is eating").	2.36±1.11	2.65±1.20	45,121.0	**0,011**
7. I feed my child myself to make him/her eat his/her food.	2.27±1.12	2.99±1.38	36,528.0	**<0.001**
8. I help my child eat the food (e.g. I cut the food into smaller pieces).	3.27±1.12	3.74±1.10	40,108.0	**<0.001**
9. I move to a different feeding area.	1.54±0.78	1.79±1.05	45,957.0	**0.013**
10. I customize the environment so that the child can eat (e.g., toys, TV, songs, videos, etc.).	1.88±1.11	2.65±1.46	36,873.5	**<0.001**
11. I prepare the food in a more interesting way (e.g. make smiling faces with the food on the plate).	1.77±0.94	1.88±0.95	48,286.0	0.146
12. I say to the child "if you don't eat, I'll be sad".	1.48±0.77	1.68±0.93	46,105.0	**0.012**
13. I say to my child "if you eat, I'll be happy".	1.72±0.99	1.83±1.00	48,063.0	0.114
14. I offer in exchange for the food a game or activity (e.g. “if you eat, you can play, we can go to the park” etc.)	2.18±1.02	2.35±1.15	48,075.0	0.141
15. I offer some other food in exchange for the meal (e.g. "if you eat, I'll give you sweet”).	2.22±1.00	2.23±1.06	51,909.0	0.967
16. I praise my child when he/she eats what I give him/her (e.g. "what a good kid, who is eating his/her beans").	3.56±1.21	3.78±1.12	46,721.0	**0.049**
17. I say something positive about the food the child is eating (e.g. "the fish is very tasty").	3.92±0.93	4.01±0.89	48,998.5	0.250
18. I explain to my child why he/she should eat (e.g. “milk is good for your health because it makes you strong”).	4.15±0.92	3.90±1.03	45,215.0	**0.009**
19. I say something to show my displeasure when the child is not eating.	2.38±1.05	2.49±0.98	48,448.0	0.184
20. I punish the child (e.g. I send him/her to his/her room).	1.26±0.59	1.25±0.52	51,513.0	0.792
21. I warn the child that I will not give him/her some food that he/she likes or that he/she will not play unless he/she eats.	2.14±1.02	2.22±1.06	50,154.5	0.486
22. I hit the kid on the hand or elsewhere on the body if he/she doesn't eat.	1.04±0.22	1.04±0.18	51,686.5	0.692
23. I have to make a physical effort to make the child eat.	1.06±0.28	1.20±0.54	46,501.0	**<0.001**

**Table 2:** Between groups comparison of the frequency distribution of the answers to each of the 23 items.

		Never	Rarely	Sometimes	Often	Always/Almost always	Chi-square	p
1. I accept that he/she may not be hungry, and I take the food away.	Healthy	2.7%	12.2%	42.2%	25.1%	17.8%	7.255	0.123
	Patients	0.7%	14.0%	36.0%	23.5%	25.7%		
2. I let the child take a break and try to feed him/her a little later.	Healthy	16.7%	29.3%	31.9%	17.4%	4.7%	12.668	**0.013**
	Patients	13.2%	27.9%	26.5%	20.6%	11.8%		
3. I urge the child to eat with prompts such as: "eat at least a little", "please try to eat", "Do you want to try them? I prepared what you like” etc.	Healthy	14.8%	24.6%	31.5%	22.0%	7.2%	8.005	0.091
	Patients	11.0%	20.6%	29.4%	25.7%	13.2%		
4. I urge the child to eat by saying for example: "the food will get cold" or "eat your beans fast" or "you can eat it" etc.	Healthy	20.7%	28.9%	32.5%	14.6%	3.3%	1.344	0.854
	Patients	21.3%	28.7%	30.1%	17.6%	2.2%		
5. I ask my family or other people to encourage the child to eat.	Healthy	53.3%	26.0%	14.4%	5.2%	1.0%	28.313	**<0.001**
	Patients	41.9%	23.5%	16.2%	12.5%	5.9%		
6. I say to my child that I or someone else in the family is eating (e.g., "I am eating," or "your brother is eating").	Healthy	28.2%	26.3%	29.2%	13.6%	2.7%	10.744	**0.030**
	Patients	22.8%	22.8%	26.5%	22.8%	5.1%		
7. I feed my child myself to make him/her eat his/her food.	Healthy	30.1%	32.5%	21.6%	12.2%	3.7%	60.127	**<0.001**
	Patients	17.6%	22.8%	21.3%	19.1%	19.1%		
8. I help my child eat the food (e.g. I cut the food into smaller pieces).	Healthy	8.8%	13.7%	32.8%	31.0%	13.7%	30.697	**<0.001**
	Patients	2.2%	14.7%	19.9%	33.8%	29.4%		
9. I move to a different feeding area	Healthy	61.6%	25.5%	10.6%	2.1%	0.3%	24.591	**<0.001**
	Patients	51.5%	30.1%	9.6%	5.1%	3.7%		
10. I customize the environment so that the child can eat (e.g., toys, TV, songs, videos, etc.).	Healthy	51.0%	24.3%	13.5%	8.0%	3.3%	54.635	**<0.001**
	Patients	33.1%	16.2%	18.4%	17.6%	14.7%		
11. I prepare the food in a more interesting way (e.g. make smiling faces with the food on the plate).	Healthy	52.0%	26.1%	15.4%	6.0%	0.4%	8.923	0.063
	Patients	44.9%	28.7%	22.1%	2.9%	1.5%		
12. I say to the child "if you don't eat, I'll be sad".	Healthy	66.8%	20.1%	11.2%	1.6%	0.3%	10.994	**0.027**
	Patients	55.9%	26.5%	12.5%	3.7%	1.5%		
13. I say to my child "if you eat, I'll be happy".	Healthy	58.6%	19.0%	15.0%	6.7%	0.8%	6.768	0.149
	Patients	49.3%	27.9%	14.0%	8.1%	0.7%		
14. I offer in exchange for the food a game or activity (e.g. “if you eat, you can play, we can go to the park” etc.)	Healthy	33.7%	26.4%	29.0%	10.3%	0.5%	8.603	0.072
	Patients	31.6%	22.8%	27.2%	16.2%	2.2%		
15. I offer some other food in exchange for the meal (e.g. "if you eat, I'll give you sweet”).	Healthy	28.8%	32.8%	27.6%	9.8%	1.0%	1.79	0.774
	Patients	30.9%	30.9%	24.3%	12.5%	1.5%		
16. I praise my child when he/she eats what I give him/her (e.g. "what a good kid, who is eating his/her beans").	Healthy	8.4%	11.4%	21.2%	34.4%	24.7%	6.158	0.188
	Patients	6.6%	59.0%	18.4%	41.2%	27.9%		
17. I say something positive about the food the child is eating (e.g. "the fish is very tasty").	Healthy	2.2%	4.4%	21.7%	42.4%	29.3%	4.194	0.380
	Patients	2.2%	3.7%	14.7%	49.3%	30.1%		
18. I explain to my child why he/she should eat (e.g. “milk is good for your health because it makes you strong”).	Healthy	2.4%	2.7%	14.4%	39.1%	41.4%	12.984	**0.011**
	Patients	5.1%	5.1%	12.5%	48.5%	28.7%		
19. I say something to show my displeasure when the child is not eating.	Healthy	23.9%	31.6%	29.9%	11.9%	2.6%	3.736	0.443
	Patients	18.4%	30.9%	36.0%	13.2%	1.5%		
20. I punish the child (e.g. I send him/her to his/her room).	Healthy	80.8%	13.3%	5.1%	0.7%	0.1%	1.881	0.758
	Patients	79.4%	16.2%	4.4%	0.0%	0.0%		
21. I warn the child that I will not give him/her some food that he/she likes or that he/she will not play unless he/she eats.	Healthy	34.1%	29.0%	26.7%	9.3%	0.9%	6.029	0.197
	Patients	29.4%	35.3%	21.3%	11.8%	2.2%		
22. I hit the kid on the hand or elsewhere on the body if he/she doesn't eat.	Healthy	97.0%	2.2%	0.8%	0.0%	0.0%	2.072	0.355
	Patients	96.3%	3.7%	0.0%	0.0%	0.0%		
23. I have to make a physical effort to make the child eat.	Healthy	95.2%	3.9%	0.8%	0.1%	0.0%	25.817	**<0.001**
	Patients	84.6%	12.5%	2.2%	0.0%	0.7%

**Table 3:** Inter-item correlation matrix (Spearman's rho). CONTROL Group (Healthy).

	q1	q2	q3	q4	q5	q6	q7	q8	q9	q10	q11	q12	q13	q14	q15	q16	q17	q18	q19	q20	q21	q22	q23
q1	^####^	.169^⁎⁎^	−0.027	−0.133^⁎⁎^	−0.168^⁎⁎^	−0.114^⁎⁎^	−0.148^⁎⁎^	.049	−0.031	−0.055	.002	−0.195^⁎⁎^	−0.202^⁎⁎^	−0.136^⁎⁎^	−0.114^⁎⁎^	−0.029	−0.011	−0.020	−0.200^⁎⁎^	−0.228^⁎⁎^	−0.225^⁎⁎^	−0.084*	−0.113^⁎⁎^
q2	.169^⁎⁎^	^####^	.312^⁎⁎^	.205^⁎⁎^	.236^⁎⁎^	.209^⁎⁎^	.286^⁎⁎^	.286^⁎⁎^	.287^⁎⁎^	.287^⁎⁎^	.209^⁎⁎^	.165^⁎⁎^	.185^⁎⁎^	.214^⁎⁎^	.195^⁎⁎^	.096^⁎⁎^	.107^⁎⁎^	.089*	.116^⁎⁎^	−0.004	.123^⁎⁎^	−0.047	.040
q3	−0.027	.312^⁎⁎^	^####^	.536^⁎⁎^	.398^⁎⁎^	.499^⁎⁎^	.455^⁎⁎^	.350^⁎⁎^	.240^⁎⁎^	.379^⁎⁎^	.180^⁎⁎^	.324^⁎⁎^	.368^⁎⁎^	.449^⁎⁎^	.439^⁎⁎^	.283^⁎⁎^	.289^⁎⁎^	.257^⁎⁎^	.403^⁎⁎^	.192^⁎⁎^	.360^⁎⁎^	.057	.103^⁎⁎^
q4	−0.133^⁎⁎^	.205^⁎⁎^	.536^⁎⁎^	^####^	.442^⁎⁎^	.495^⁎⁎^	.429^⁎⁎^	.272^⁎⁎^	.186^⁎⁎^	.288^⁎⁎^	.127^⁎⁎^	.388^⁎⁎^	.411^⁎⁎^	.412^⁎⁎^	.412^⁎⁎^	.248^⁎⁎^	.271^⁎⁎^	.213^⁎⁎^	.452^⁎⁎^	.259^⁎⁎^	.417^⁎⁎^	.094^⁎⁎^	.166^⁎⁎^
q5	−0.168^⁎⁎^	.236^⁎⁎^	.398^⁎⁎^	.442^⁎⁎^	^####^	.524^⁎⁎^	.435^⁎⁎^	.284^⁎⁎^	.317^⁎⁎^	.334^⁎⁎^	.203^⁎⁎^	.356^⁎⁎^	.358^⁎⁎^	.394^⁎⁎^	.311^⁎⁎^	.164^⁎⁎^	.192^⁎⁎^	.090*	.409^⁎⁎^	.220^⁎⁎^	.326^⁎⁎^	.102^⁎⁎^	.173^⁎⁎^
q6	−0.114^⁎⁎^	.209^⁎⁎^	.499^⁎⁎^	.495^⁎⁎^	.524^⁎⁎^	^####^	.442^⁎⁎^	.303^⁎⁎^	.288^⁎⁎^	.322^⁎⁎^	.147^⁎⁎^	.406^⁎⁎^	.426^⁎⁎^	.436^⁎⁎^	.370^⁎⁎^	.302^⁎⁎^	.288^⁎⁎^	.204^⁎⁎^	.399^⁎⁎^	.214^⁎⁎^	.343^⁎⁎^	.097^⁎⁎^	.148^⁎⁎^
q7	−0.148^⁎⁎^	.286^⁎⁎^	.455^⁎⁎^	.429^⁎⁎^	.435^⁎⁎^	.442^⁎⁎^	^####^	.435^⁎⁎^	.376^⁎⁎^	.519^⁎⁎^	.172^⁎⁎^	.362^⁎⁎^	.358^⁎⁎^	.465^⁎⁎^	.397^⁎⁎^	.143^⁎⁎^	.231^⁎⁎^	.117^⁎⁎^	.406^⁎⁎^	.189^⁎⁎^	.358^⁎⁎^	.105^⁎⁎^	.243^⁎⁎^
q8	.049	.286^⁎⁎^	.350^⁎⁎^	.272^⁎⁎^	.284^⁎⁎^	.303^⁎⁎^	.435^⁎⁎^	^####^	.297^⁎⁎^	.362^⁎⁎^	.133^⁎⁎^	.236^⁎⁎^	.236^⁎⁎^	.325^⁎⁎^	.285^⁎⁎^	.179^⁎⁎^	.287^⁎⁎^	.145^⁎⁎^	.269^⁎⁎^	.095^⁎⁎^	.201^⁎⁎^	.037	.115^⁎⁎^
q9	−0.031	.287^⁎⁎^	.240^⁎⁎^	.186^⁎⁎^	.317^⁎⁎^	.288^⁎⁎^	.376^⁎⁎^	.297^⁎⁎^	^####^	.460^⁎⁎^	.277^⁎⁎^	.244^⁎⁎^	.272^⁎⁎^	.279^⁎⁎^	.289^⁎⁎^	.046	.033	.030	.175^⁎⁎^	.091*	.146^⁎⁎^	.058	.168^⁎⁎^
q10	−0.055	.287^⁎⁎^	.379^⁎⁎^	.288^⁎⁎^	.334^⁎⁎^	.322^⁎⁎^	.519^⁎⁎^	.362^⁎⁎^	.460^⁎⁎^	^####^	.282^⁎⁎^	.324^⁎⁎^	.334^⁎⁎^	.422^⁎⁎^	.367^⁎⁎^	.173^⁎⁎^	.139^⁎⁎^	.072*	.292^⁎⁎^	.133^⁎⁎^	.238^⁎⁎^	.111^⁎⁎^	.185^⁎⁎^
q11	.002	.209^⁎⁎^	.180^⁎⁎^	.127^⁎⁎^	.203^⁎⁎^	.147^⁎⁎^	.172^⁎⁎^	.133^⁎⁎^	.277^⁎⁎^	.282^⁎⁎^	^####^	.202^⁎⁎^	.226^⁎⁎^	.192^⁎⁎^	.149^⁎⁎^	.113^⁎⁎^	.108^⁎⁎^	.125^⁎⁎^	.117^⁎⁎^	.025	.050	.004	−0.004
q12	−0.195^⁎⁎^	.165^⁎⁎^	.324^⁎⁎^	.388^⁎⁎^	.356^⁎⁎^	.406^⁎⁎^	.362^⁎⁎^	.236^⁎⁎^	.244^⁎⁎^	.324^⁎⁎^	.202^⁎⁎^	^####^	.769^⁎⁎^	.438^⁎⁎^	.346^⁎⁎^	.246^⁎⁎^	.185^⁎⁎^	.156^⁎⁎^	.468^⁎⁎^	.275^⁎⁎^	.335^⁎⁎^	.086*	.186^⁎⁎^
q13	−0.202^⁎⁎^	.185^⁎⁎^	.368^⁎⁎^	.411^⁎⁎^	.358^⁎⁎^	.426^⁎⁎^	.358^⁎⁎^	.236^⁎⁎^	.272^⁎⁎^	.334^⁎⁎^	.226^⁎⁎^	.769^⁎⁎^	^####^	.490^⁎⁎^	.376^⁎⁎^	.284^⁎⁎^	.195^⁎⁎^	.178^⁎⁎^	.436^⁎⁎^	.224^⁎⁎^	.313^⁎⁎^	.071	.174^⁎⁎^
q14	−0.136^⁎⁎^	.214^⁎⁎^	.449^⁎⁎^	.412^⁎⁎^	.394^⁎⁎^	.436^⁎⁎^	.465^⁎⁎^	.325^⁎⁎^	.279^⁎⁎^	.422^⁎⁎^	.192^⁎⁎^	.438^⁎⁎^	.490^⁎⁎^	^####^	.640^⁎⁎^	.341^⁎⁎^	.248^⁎⁎^	.169^⁎⁎^	.432^⁎⁎^	.237^⁎⁎^	.486^⁎⁎^	.081*	.148^⁎⁎^
q15	−0.114^⁎⁎^	.195^⁎⁎^	.439^⁎⁎^	.412^⁎⁎^	.311^⁎⁎^	.370^⁎⁎^	.397^⁎⁎^	.285^⁎⁎^	.289^⁎⁎^	.367^⁎⁎^	.149^⁎⁎^	.346^⁎⁎^	.376^⁎⁎^	.640^⁎⁎^	^####^	.277^⁎⁎^	.210^⁎⁎^	.150^⁎⁎^	.390^⁎⁎^	.253^⁎⁎^	.587^⁎⁎^	.054	.111^⁎⁎^
q16	−0.029	.096^⁎⁎^	.283^⁎⁎^	.248^⁎⁎^	.164^⁎⁎^	.302^⁎⁎^	.143^⁎⁎^	.179^⁎⁎^	.046	.173^⁎⁎^	.113^⁎⁎^	.246^⁎⁎^	.284^⁎⁎^	.341^⁎⁎^	.277^⁎⁎^	^####^	.584^⁎⁎^	.397^⁎⁎^	.296^⁎⁎^	.102^⁎⁎^	.262^⁎⁎^	.034	.012
q17	−0.011	.107^⁎⁎^	.289^⁎⁎^	.271^⁎⁎^	.192^⁎⁎^	.288^⁎⁎^	.231^⁎⁎^	.287^⁎⁎^	.033	.139^⁎⁎^	.108^⁎⁎^	.185^⁎⁎^	.195^⁎⁎^	.248^⁎⁎^	.210^⁎⁎^	.584^⁎⁎^	^####^	.606^⁎⁎^	.322^⁎⁎^	.062	.243^⁎⁎^	−0.017	−0.001
q18	−0.020	.089*	.257^⁎⁎^	.213^⁎⁎^	.090*	.204^⁎⁎^	.117^⁎⁎^	.145^⁎⁎^	.030	.072*	.125^⁎⁎^	.156^⁎⁎^	.178^⁎⁎^	.169^⁎⁎^	.150^⁎⁎^	.397^⁎⁎^	.606^⁎⁎^	^####^	.267^⁎⁎^	.030	.199^⁎⁎^	−0.067	−0.032
q19	−0.200^⁎⁎^	.116^⁎⁎^	.403^⁎⁎^	.452^⁎⁎^	.409^⁎⁎^	.399^⁎⁎^	.406^⁎⁎^	.269^⁎⁎^	.175^⁎⁎^	.292^⁎⁎^	.117^⁎⁎^	.468^⁎⁎^	.436^⁎⁎^	.432^⁎⁎^	.390^⁎⁎^	.296^⁎⁎^	.322^⁎⁎^	.267^⁎⁎^	^####^	.317^⁎⁎^	.514^⁎⁎^	.119^⁎⁎^	.129^⁎⁎^
q20	−0.228^⁎⁎^	−0.004	.192^⁎⁎^	.259^⁎⁎^	.220^⁎⁎^	.214^⁎⁎^	.189^⁎⁎^	.095^⁎⁎^	.091*	.133^⁎⁎^	.025	.275^⁎⁎^	.224^⁎⁎^	.237^⁎⁎^	.253^⁎⁎^	.102^⁎⁎^	.062	.030	.317^⁎⁎^	^####^	.395^⁎⁎^	.257^⁎⁎^	.206^⁎⁎^
q21	−0.225^⁎⁎^	.123^⁎⁎^	.360^⁎⁎^	.417^⁎⁎^	.326^⁎⁎^	.343^⁎⁎^	.358^⁎⁎^	.201^⁎⁎^	.146^⁎⁎^	.238^⁎⁎^	.050	.335^⁎⁎^	.313^⁎⁎^	.486^⁎⁎^	.587^⁎⁎^	.262^⁎⁎^	.243^⁎⁎^	.199^⁎⁎^	.514^⁎⁎^	.395^⁎⁎^	^####^	.141^⁎⁎^	.148^⁎⁎^
q22	−0.084*	−0.047	.057	.094^⁎⁎^	.102^⁎⁎^	.097^⁎⁎^	.105^⁎⁎^	.037	.058	.111^⁎⁎^	.004	.086*	.071	.081*	.054	.034	−0.017	−0.067	.119^⁎⁎^	.257^⁎⁎^	.141^⁎⁎^	^####^	.182^⁎⁎^
q23	−0.113^⁎⁎^	.040	.103^⁎⁎^	.166^⁎⁎^	.173^⁎⁎^	.148^⁎⁎^	.243^⁎⁎^	.115^⁎⁎^	.168^⁎⁎^	.185^⁎⁎^	−0.004	.186^⁎⁎^	.174^⁎⁎^	.148^⁎⁎^	.111^⁎⁎^	.012	−0.001	−0.032	.129^⁎⁎^	.206^⁎⁎^	.148^⁎⁎^	.182^⁎⁎^	^####^

**Correlation is significant at the 0.01 level (2-tailed).

*Correlation is significant at the 0.05 level (2-tailed).

####Correlation coefficient = 1.

**Table 4:** Inter-item correlation matrix (Spearman's rho). CLINICAL Group (With a gastrointestinal disease).

	q1	q2	q3	q4	q5	q6	q7	q8	q9	q10	q11	q12	q13	q14	q15	q16	q17	q18	q19	q20	q21	q22	q23
q1	^####^	.257^⁎⁎^	−0.130	−0.271^⁎⁎^	−0.250^⁎⁎^	−0.299^⁎⁎^	−0.146	.046	−0.156	−0.112	−0.053	−0.276^⁎⁎^	−0.178*	−0.205*	−0.305^⁎⁎^	−0.011	−0.027	.051	−0.235^⁎⁎^	−0.201*	−0.183*	−0.080	−0.028
q2	.257^⁎⁎^	^####^	.133	−0.037	.116	.112	.173*	.239^⁎⁎^	.189*	.201*	.180*	.032	.098	.066	.021	.117	.130	.063	−0.002	−0.053	.019	−0.056	.105
q3	−0.130	.133	^####^	.380^⁎⁎^	.629^⁎⁎^	.532^⁎⁎^	.409^⁎⁎^	.348^⁎⁎^	.314^⁎⁎^	.390^⁎⁎^	.099	.117	.104	.542^⁎⁎^	.373^⁎⁎^	.300^⁎⁎^	.263^⁎⁎^	.113	.395^⁎⁎^	.060	.257^⁎⁎^	.044	.149
q4	−0.271^⁎⁎^	−0.037	.380^⁎⁎^	^####^	.398^⁎⁎^	.419^⁎⁎^	.212*	.139	.158	.142	.054	.345^⁎⁎^	.392^⁎⁎^	.455^⁎⁎^	.383^⁎⁎^	.185*	.230^⁎⁎^	.082	.425^⁎⁎^	.192*	.356^⁎⁎^	.139	.105
q5	−0.250^⁎⁎^	.116	.629^⁎⁎^	.398^⁎⁎^	^####^	.506^⁎⁎^	.351^⁎⁎^	.257^⁎⁎^	.327^⁎⁎^	.313^⁎⁎^	.140	.261^⁎⁎^	.307^⁎⁎^	.491^⁎⁎^	.316^⁎⁎^	.244^⁎⁎^	.221^⁎⁎^	−0.013	.365^⁎⁎^	.051	.214*	.118	.105
q6	−0.299^⁎⁎^	.112	.532^⁎⁎^	.419^⁎⁎^	.506^⁎⁎^	^####^	.383^⁎⁎^	.382^⁎⁎^	.330^⁎⁎^	.453^⁎⁎^	.050	.414^⁎⁎^	.420^⁎⁎^	.555^⁎⁎^	.506^⁎⁎^	.406^⁎⁎^	.265^⁎⁎^	.153	.352^⁎⁎^	.194*	.320^⁎⁎^	−0.005	.075
q7	−0.146	.173*	.409^⁎⁎^	.212*	.351^⁎⁎^	.383^⁎⁎^	^####^	.640^⁎⁎^	.311^⁎⁎^	.596^⁎⁎^	.056	.074	.143	.389^⁎⁎^	.196*	.173*	.096	−0.037	.319^⁎⁎^	.152	.202*	.032	.256^⁎⁎^
q8	.046	.239^⁎⁎^	.348^⁎⁎^	.139	.257^⁎⁎^	.382^⁎⁎^	.640^⁎⁎^	^####^	.212*	.554^⁎⁎^	.064	.121	.163	.218*	.070	.311^⁎⁎^	.210*	.042	.239^⁎⁎^	.058	.019	−0.092	.201*
q9	−0.156	.189*	.314^⁎⁎^	.158	.327^⁎⁎^	.330^⁎⁎^	.311^⁎⁎^	.212*	^####^	.446^⁎⁎^	.151	.049	.053	.345^⁎⁎^	.204*	.208*	.104	−0.124	.139	.051	−0.012	.002	.248^⁎⁎^
q10	−0.112	.201*	.390^⁎⁎^	.142	.313^⁎⁎^	.453^⁎⁎^	.596^⁎⁎^	.554^⁎⁎^	.446^⁎⁎^	^####^	.124	.216*	.205*	.356^⁎⁎^	.294^⁎⁎^	.250^⁎⁎^	.118	−0.107	.270^⁎⁎^	.286^⁎⁎^	.180*	.108	.251^⁎⁎^
q11	−0.053	.180*	.099	.054	.140	.050	.056	.064	.151	.124	^####^	.140	.124	.004	.025	−0.169*	.091	.169*	−0.009	.047	.029	−0.040	.082
q12	−0.276^⁎⁎^	.032	.117	.345^⁎⁎^	.261^⁎⁎^	.414^⁎⁎^	.074	.121	.049	.216*	.140	^####^	.774^⁎⁎^	.283^⁎⁎^	.417^⁎⁎^	.111	.115	.010	.321^⁎⁎^	.236^⁎⁎^	.202*	.049	.037
q13	−0.178*	.098	.104	.392^⁎⁎^	.307^⁎⁎^	.420^⁎⁎^	.143	.163	.053	.205*	.124	.774^⁎⁎^	^####^	.379^⁎⁎^	.357^⁎⁎^	.194*	.159	.093	.380^⁎⁎^	.222^⁎⁎^	.316^⁎⁎^	.040	.100
q14	−0.205*	.066	.542^⁎⁎^	.455^⁎⁎^	.491^⁎⁎^	.555^⁎⁎^	.389^⁎⁎^	.218*	.345^⁎⁎^	.356^⁎⁎^	.004	.283^⁎⁎^	.379^⁎⁎^	^####^	.570^⁎⁎^	.350^⁎⁎^	.279^⁎⁎^	.092	.377^⁎⁎^	.245^⁎⁎^	.513^⁎⁎^	.122	.187*
q15	−0.305^⁎⁎^	.021	.373^⁎⁎^	.383^⁎⁎^	.316^⁎⁎^	.506^⁎⁎^	.196*	.070	.204*	.294^⁎⁎^	.025	.417^⁎⁎^	.357^⁎⁎^	.570^⁎⁎^	^####^	.208*	.225^⁎⁎^	.112	.320^⁎⁎^	.309^⁎⁎^	.559^⁎⁎^	.116	−0.078
q16	−0.011	.117	.300^⁎⁎^	.185*	.244^⁎⁎^	.406^⁎⁎^	.173*	.311^⁎⁎^	.208*	.250^⁎⁎^	−0.169*	.111	.194*	.350^⁎⁎^	.208*	^####^	.488^⁎⁎^	.083	.168	.009	.212*	−0.032	−0.002
q17	−0.027	.130	.263^⁎⁎^	.230^⁎⁎^	.221^⁎⁎^	.265^⁎⁎^	.096	.210*	.104	.118	.091	.115	.159	.279^⁎⁎^	.225^⁎⁎^	.488^⁎⁎^	^####^	.317^⁎⁎^	.299^⁎⁎^	.094	.312^⁎⁎^	−0.024	−0.097
q18	.051	.063	.113	.082	−0.013	.153	−0.037	.042	−0.124	−0.107	.169*	.010	.093	.092	.112	.083	.317^⁎⁎^	^####^	.076	−0.092	.232^⁎⁎^	−0.181*	−0.152
q19	−0.235^⁎⁎^	−0.002	.395^⁎⁎^	.425^⁎⁎^	.365^⁎⁎^	.352^⁎⁎^	.319^⁎⁎^	.239^⁎⁎^	.139	.270^⁎⁎^	−0.009	.321^⁎⁎^	.380^⁎⁎^	.377^⁎⁎^	.320^⁎⁎^	.168	.299^⁎⁎^	.076	^####^	.253^⁎⁎^	.334^⁎⁎^	.075	.149
q20	−0.201*	−0.053	.060	.192*	.051	.194*	.152	.058	.051	.286^⁎⁎^	.047	.236^⁎⁎^	.222^⁎⁎^	.245^⁎⁎^	.309^⁎⁎^	.009	.094	−0.092	.253^⁎⁎^	^####^	.358^⁎⁎^	.177*	.183*
q21	−0.183*	.019	.257^⁎⁎^	.356^⁎⁎^	.214*	.320^⁎⁎^	.202*	.019	−0.012	.180*	.029	.202*	.316^⁎⁎^	.513^⁎⁎^	.559^⁎⁎^	.212*	.312^⁎⁎^	.232^⁎⁎^	.334^⁎⁎^	.358^⁎⁎^	^####^	.146	.038
q22	−0.080	−0.056	.044	.139	.118	−0.005	.032	−0.092	.002	.108	−0.040	.049	.040	.122	.116	−0.032	−0.024	−0.181*	.075	.177*	.146	^####^	.351^⁎⁎^
q23	−0.028	.105	.149	.105	.105	.075	.256^⁎⁎^	.201*	.248^⁎⁎^	.251^⁎⁎^	.082	.037	.100	.187*	−0.078	−0.002	−0.097	−0.152	.149	.183*	.038	.351^⁎⁎^	^####^

**Correlation is significant at the 0.01 level (2-tailed).

*Correlation is significant at the 0.05 level (2-tailed).

####Correlation coefficient = 1.

## 2 Experimental design, materials and methods

Parental feeding practices significantly influence child eating behavior [2], [3], [4], [5], [6], [7], [8], [9]. The data presented was obtained through a cross-sectional case control study that aimed to record parental practices to manage feeding problems in children with typical development and children with gastrointestinal diseases. A detailed methodology is provided elsewhere [2]. In brief, a 23-item (5-point Likert type) questionnaire was used to assess parental practices during feeding in two samples. A clinical one (children with a gastrointestinal disease) and a sample of healthy children (control group). All children were aged between one and seven years old. After obtaining approval by the Ministry of Education of Greece normative sample was collected from 75 kindergartens from various geographic regions of Greece via a convenient sample strategy. Sampling was based on representativeness of a large geographical area of Greece, including both urban and rural areas. Head teachers that agreed to participate in the study administered the set of questions to the parents. Any parent who was willing to participate filled out the questions and returned them to the head teachers. Potential participants in the clinical group were approached through the outpatient unit of a gastroenterology department. A total of 765 healthy children and 136 children with gastrointestinal diseases participated in the study. Besides the descriptive measures, Mann-Whitney U and chi-square tests were conducted and Spearman's rho correlation coefficients were calculated. For the purposes of the analysis SPSS v.20 (IBM, 142 Armonk, New York, USA) was used. Statistical significance level was set at 0.05.

## Declaration of Competing Interest

The authors declare that they have no known competing financial interests or personal relationships which have, or could be perceived to have, influenced the work reported in this article.

## Appendix Supplementary materials

Image, application 1
