# Supplementary material for: Parental feeding practices data in healthy children and children with gastrointestinal diseases
Source: Data Brief. 2020 Jul 17;31:106036. doi: 10.1016/j.dib.2020.106036 (PMC7381492; doi:10.1016/j.dib.2020.106036)
Supplement: Supplementary file 1 [file mmc1.doc]

| **Appendix A. The set of questions** | | | | | |
| --- | --- | --- | --- | --- | --- |
| **Feeding problem management practices** | **How often do you react in the following ways when your child refuses to eat or eats small quantities. Tick the best answer.** | | | | |
| **1. I accept that he/she may not be hungry, and I take the food away.** |  Never |  Rarely |  Sometimes |  Often |  Always |
| **2. I let the child take a break and try to feed him/her a little later.** |  Never |  Rarely |  Sometimes |  Often |  Always |
| **3. I urge the child to eat with prompts such as: "eat at least a little", "please try to eat", "Do you want to try them? I prepared what you like” etc.** |  Never |  Rarely |  Sometimes |  Often |  Always |
| **4. I urge the child to eat by saying for example: "the food will get cold" or "eat your beans fast" or "you can eat it" etc.** |  Never |  Rarely |  Sometimes |  Often |  Always |
| **5. I ask my family or other people to encourage the child to eat.** |  Never |  Rarely |  Sometimes |  Often |  Always |
| **6. I say to my child that I or someone else in the family is eating (e.g., "I am eating," or "your brother is eating").** |  Never |  Rarely |  Sometimes |  Often |  Always |
| **7. I feed my child myself to make him/her eat his/her food.** |  Never |  Rarely |  Sometimes |  Often |  Always |
| **8. I help my child eat the food (e.g. I cut the food into smaller pieces).** |  Never |  Rarely |  Sometimes |  Often |  Always |
| **9. I move to a different feeding area** |  Never |  Rarely |  Sometimes |  Often |  Always |
| **10. I customize the environment so that the child can eat (e.g., toys, TV, songs, videos, etc.).** |  Never |  Rarely |  Sometimes |  Often |  Always |
| **11. I prepare the food in a more interesting way (e.g. make smiling faces with the food on the plate).** |  Never |  Rarely |  Sometimes |  Often |  Always |
| **12. I say to the child "if you don't eat, I'll be sad".** |  Never |  Rarely |  Sometimes |  Often |  Always |
| **13. I say to my child "if you eat, I'll be happy".** |  Never |  Rarely |  Sometimes |  Often |  Always |
| **14. I offer in exchange for the food a game or activity (e.g. “if you eat, you can play, we can go to the park” etc.)** |  Never |  Rarely |  Sometimes |  Often |  Always |
| **15. I offer some other food in exchange for the meal (e.g. "if you eat, I'll give you sweet”).** |  Never |  Rarely |  Sometimes |  Often |  Always |
| **16. I praise my child when he/she eats what I give him/her (e.g. "what a good kid, who is eating his/her beans").** |  Never |  Rarely |  Sometimes |  Often |  Always |
| **17. I say something positive about the food the child is eating (e.g. "the fish is very tasty").** |  Never |  Rarely |  Sometimes |  Often |  Always |
| **18. I explain to my child why he/she should eat (e.g. “milk is good for your health because it makes you strong”).** |  Never |  Rarely |  Sometimes |  Often |  Always |
| **19. I say something to show my displeasure when the child is not eating.** |  Never |  Rarely |  Sometimes |  Often |  Always |
| **20. I punish the child (e.g. I send him/her to his/her room).** |  Never |  Rarely |  Sometimes |  Often |  Always |
| **21. I warn the child that I will not give him/her some food that he/she likes or that he/she will not play unless he/she eats.** |  Never |  Rarely |  Sometimes |  Often   Often |  Always |
| **22. I hit the kid on the hand or elsewhere on the body if he/she doesn't eat.** |  Never |  Rarely |  Sometimes |  Often |  Always |
| **23. I have to make a physical effort to make the child eat.** |  Never |  Rarely |  Sometimes |  Often |  Always |
